# Adaptive responses of plants to light stress: mechanisms of photoprotection and acclimation. A review

**DOI:** 10.3389/fpls.2025.1550125

**Published:** 2025-03-28

**Authors:** Imran Khan, Sohail, Shah Zaman, Guihua Li, Mei Fu

**Affiliations:** ^1^ Guangdong Key Laboratory for New Technology Research of Vegetables, Vegetable Research Institute, Guangdong Academy of Agricultural Sciences, Guangzhou, China; ^2^ College of Bioscience and Biotechnology, Yangzhou University, Jiangsu, Yangzhou, China; ^3^ Department of Botany, University of Malakand KPK, Chakdara, Pakistan

**Keywords:** climate change, light, reactive oxygen species, biogenesis of PSII, photosystem II repair cycle

## Abstract

Plants depend on solar energy for growth via oxygenic photosynthesis. However, when light levels exceed the optimal range for photosynthesis, it causes abiotic stress and harms plant physiology. In response to excessive light, plants activate a series of signaling pathways starting from the chloroplast and affecting the entire plant, leading to stress-specific physiological changes. These signals prompt various physiological and biochemical adjustments aimed at counteracting the negative impacts of high light intensity, including photodamage and photoinhibition. Mechanisms to protect against light stress involve scavenging of chloroplastic reactive oxygen species (ROS), adjustments in chloroplast and stomatal positioning, and increased anthocyanin production to safeguard the photosynthetic machinery. Given that this machinery is a primary target for stress-induced damage, plants have evolved acclimation strategies like dissipating thermal energy via non-photochemical quenching (NPQ), repairing Photosystem II (PSII), and regulating the transcription of photosynthetic proteins. Fluctuating light presents a less severe but consistent stress, which has not been extensively studied. Nevertheless, current research indicates that state transitions and cyclic electron flow play crucial roles in helping plants adapt to varying light conditions. This review encapsulates the latest understanding of plant physiological and biochemical responses to both high light and low light stress.

## 1 Introduction

Light exhibits both particle and wave properties. A photon refers to a light particle, and the energy it carries is termed as quantum. The intensity or quantity of light is defined as the rate at which light disperses over a specific surface area. This intensity is also known as the amount of energy that is transferred per unit area (Taiz and Zeiger, 2006; Blankenship, 2021). One of the most important parameters in measuring light intensity is the quality of the spectral region, the quantity, and the direction (Both et al., 2015). The amount of solar energy that falls on an area of a flat leaf has an irradiance value measured in units of solar energy per unit of time falling on that area. The amount of light a surface receives per unit time is termed photon irradiance. The ability of a plant to utilize light efficiently depends on the angle at which light strikes its surface during photosynthesis. Light can be absorbed directly or indirectly, influencing plant growth and productivity (Schirillo, 2013). The omnidirectional measurement, which is able to capture light from all directions at the same time, is called the fluence rate (Rupert and Latarjet, 1978). Typically, light is intercepted by shoots, whole plants, and chloroplasts from multiple angles rather than a single direction, increasing photosynthetic efficiency due to even light distribution (Schirillo, 2013). Optimizing light absorption and maximizing photosynthetic potential are essential for plant growth and productivity.

While light serves as a primary energy source for photosynthesis, it also acts as a critical environmental factor that influences plant development and survival. However, fluctuation in light intensity poses significant challenge to plants, leading to light stress. Light stress occurs when the light condition deviates from the optimal level, either through excessive light exposure high light stress are insufficient light availability low light stress (Fiorucci and Fankhauser, 2017). High light intensity can cause photo damage, reducing photosynthetic efficiency in leading to the formation of ROS which can damage cellular structure (Khan et al., 2023). On the other hand, low light intensity limits energy capture, restricting plant growth and development. In addition, rapidly changing light condition further challenge the photosynthetic machinery, requiring plants to dynamically adjust their photosynthetic processes (Tang et al., 2023). To cope with light stress, plants have evolved various adaptive mechanisms to regulate light absorption and minimize photodamage. These include the production and scavenging of chloroplastic ROS, chloroplast movement, stomatal regulation, and the synthesis of photoprotective pigments such as anthocyanin (Yang et al., 2019). Additionally, systematic signaling pathways help coordinate these responses. Given the sensitivity of photosynthesis to light fluctuation, plants modulate the structure and function of the liquid membrane bounded protein complexes to optimize energy use and protect the photosynthetic apparatus from damage (Pottosin and Shabala, 2016).

Plants have evolved a variety of photoprotective mechanisms to deal with high and low Light levels, which ensures the stability of their photosynthetic apparatus. While much progress has been made in understanding individual photoprotective pathways, new findings indicate complex interactions between various mechanisms that are still poorly understood. Furthermore, it is unclear how these mechanisms work together to promote plant resilience in a variety of environments. This review seeks to close the gap by synthesizing recent advances in photoprotection research, with a particular emphasis on how multiple photoprotective strategies are integrated at the molecular and physiological levels. First, we discuss the key mechanisms of photoprotection, such as non-photochemical quenching, antioxidant defense systems, and chloroplast movement. Next, we explore how hormonal and transcriptional networks control light stresses responses. Lastly, we highlight potential applications of photoprotection research to improve plant resilience in natural and agricultural environments. In changing environmental scenarios, understanding these interactions is essential for optimizing growth conditions and enhancing crop productivity.

## 2 Effect of light intensities on plant growth and development

**Figure 1:**
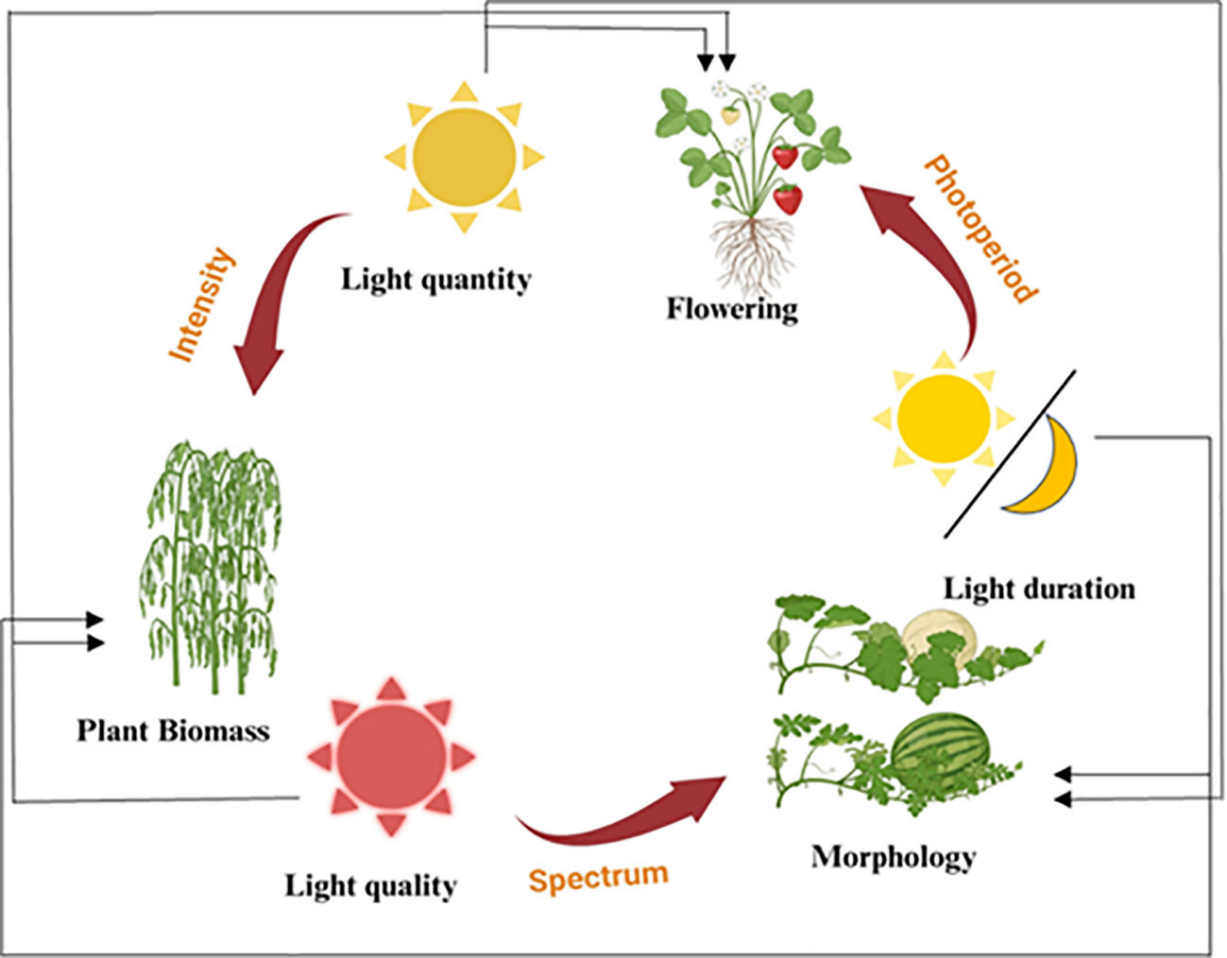
The influence of various light factors on plant growth stages and characteristics. This diagram illustrates how different aspects of light, including light quantity, light quality, and light duration, affect various stages of plant development and morphology. Light quantity (intensity) impacts the overall plant biomass. On the other hand, light quality (spectrum) affects plant morphology, including the development of specific physical traits. Light duration (photoperiod) regulates flowering and other photoperiod-sensitive processes in plants. The cycle demonstrates the interconnected nature of light factors and their cumulative effects on plant growth and development.

Light intensity and wavelength are two of the most important external parameters affecting plant physiology and biochemistry (Yang et al., 2018a). Even a slight change in light intensity can significantly alter leaf structure and metabolism in many crops (Wu et al., 2017). Under low light conditions, root, stem, and leaf development (dry matter), as well as plant growth as a whole, is often restricted (**Figure 1**). Low light conditions result in decreased transpiration and stomatal conductance (Yang et al., 2014, Yang et al., 2017). Due to reduced stomatal conductance, carbon dioxide (CO_2_) cannot diffuse into the leaf, reducing the availability of CO_2_ for the calvin cycle, and ultimately reducing photosynthesis (Irfan et al., 2019). The leaves of crops grown in low light conditions are smaller and thinner than those grown in high light conditions (Wu et al., 2017). Agricultural productivity is reduced due to shading, increasing plant height and lodging rate, and inhibiting the circulation of nutrients, water, and photosynthetic products. It has been shown that light intensity is a key predictor of fundamental plant functions such as germination, leaf growth, photosynthesis, bud expansion, flower initiation, and cell division (Kong et al., 2016; Wu et al., 2018; Yang et al., 2018b). However, even modest increases in light intensity can have significant physiological and developmental effects. In general, higher light levels promote photosynthesis and plant growth, however, excessive light can lead to photoinhibition, oxidative stress, and impaired photosynthetic efficiency (Yang et al., 2015; Wu et al., 2016). When light intensity is too high than the optimal range for photosynthesis, it causes abiotic stress and physiological damage to the plants (Singh and Thakur, 2018). In response to high light stress, plants start a series of signal transduction from chloroplasts to whole cells and from locally stressed tissues to the rest of the plant body. These signals set off several physiological and biochemical processes that are meant to counteract the harmful effects of intense light, like photoinhibition and photodamage (Horton, 2012). Light stress protection mechanisms include chloroplastic ROS scavenging, stomatal movement, chloroplast, and anthocyanin production (Shi et al., 2022). The chloroplasts of plants use solar energy to convert CO_2_ and water into organic matter and oxygen. Since light intensity and spectral quality vary across time and space, light serves as a source of energy for photosynthesis and is a crucial environmental factor. When light isn’t good for plant growth, it can become a significant abiotic stressor (Fiorucci and Fankhauser, 2017).

Low light intensity is insufficient for plant growth. On the contrary, high light intensity is known to be photodamaging to plants. Furthermore, changes in light intensity can reduce photosynthetic efficiency. During low light conditions, photon availability is reduced, limiting photosynthesis and carbon uptake. In contrast, excessive light can cause photoinhibition by overexciting the photosynthetic apparatus, producing ROS, and the damaging photosystem (PSII), ultimately decreasing photosynthetic efficiency- (Walters, 2005). In response to high light stress, plants have evolved various protective mechanisms such as chloroplast ROS scavenging and movement, stomatal opening or closure (based on the severity of light), and anthocyanin accumulation in coordination with systemic signaling. By regulating stomatal opening and closing, chloroplastic ROS are produced and scavenged by the plant’s photoprotective mechanisms, and systemic signaling coordinates the synthesis of anthocyanins (Pottosin and Shabala, 2016).

## 3 Systemic responses of plants to light stress

Excessive light absorption could boost the population of excited, highly reactive intermediates after photosynthesis and create a huge potential for biphase light damage to plants (Bassi and Dall’osto, 2021). Plants use several photoprotective strategies and molecules as counter-measurements to repair the damage done by light stress. A few of these are transcription factors, phytohormones, scavengers ROS as well as mitogen-activated protein kinases. This can occur by decreasing the content of redox-active molecules, reducing light absorption, or reprogramming the metabolism and transcriptome including modifications at several steps (Bassi and Dall’osto, 2021; Sánchez-Mcsweeney et al., 2021).

## 4 Chloroplast movement in response to light

The movement of chloroplasts in response to light conditions is a key adaptation that enables plants to optimize photosynthesis while minimizing the adverse effects of excessive light exposure. The chloroplasts can strategically position themselves in fluctuating light environments, such as those found beneath the tree canopy, and respond to changing light conditions through two key strategies: they maximize light absorption during low light (known as the accumulation response) and minimize damage by moving away from the light source during high light conditions (known as the avoidance response) (Wada and Kong, 2011). Chloroplasts move towards illuminated regions most under low light to optimize their distribution over the cell surface for better efficiency of photosynthesis. Nevertheless, under high light conditions, the chloroplasts move to positions that are perpendicular to the surface of the cell walls and embed in their sides moving sidewise at a right angle direction thus avoiding high light on them (Cazzaniga et al., 2013). This behavior maximizes photosynthetic activity under low-light conditions and protects against potential photodamage under high light conditions by relocating the cellular components to other loci within the cell. This mechanism is not only effective in preventing damage but also enhances cellular adoptability. If adjustments are not well done, it may die with a damaged leaf and ruptured chloroplast (Kasahara et al., 2002).

**Figure 2:**
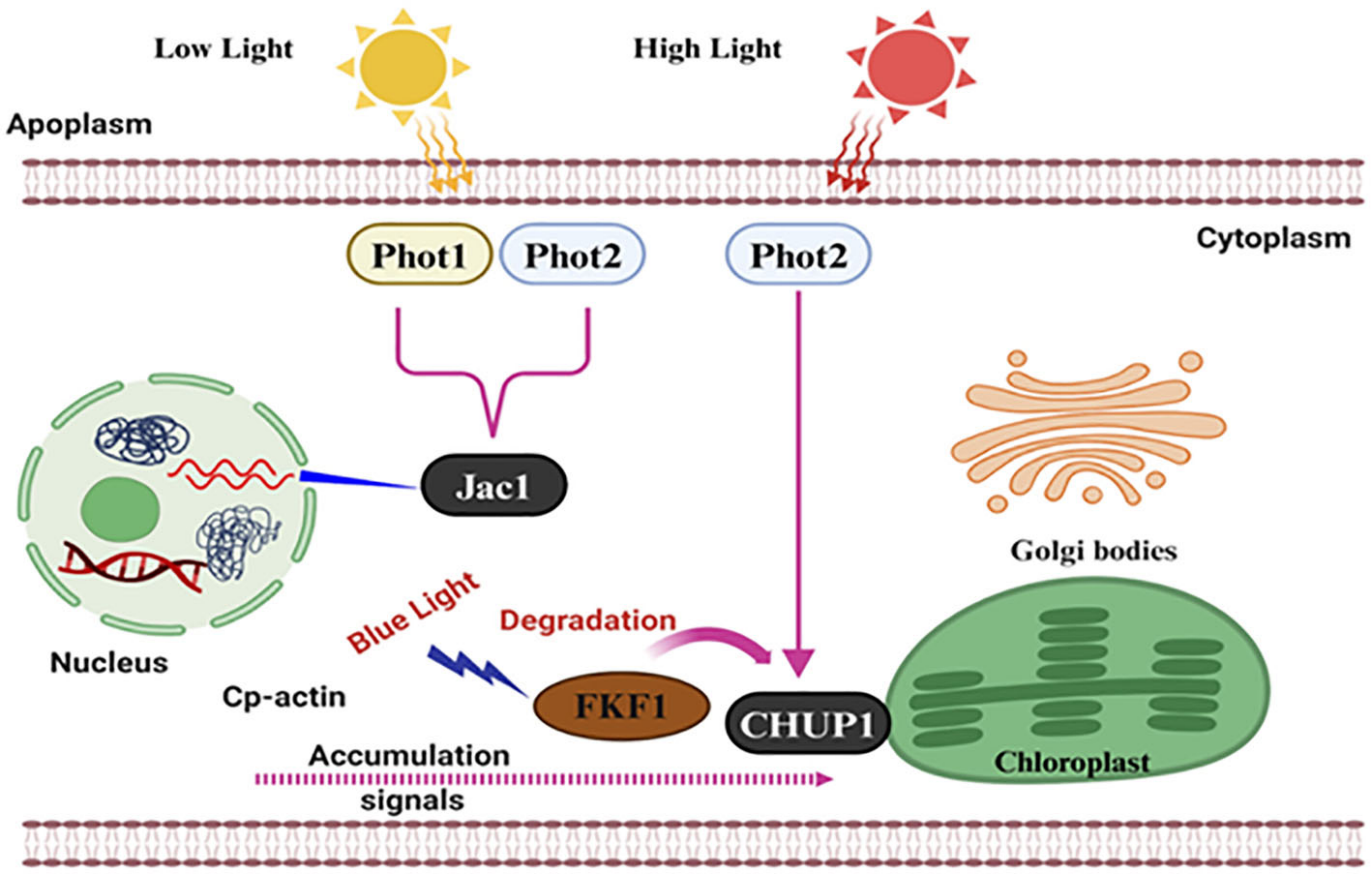
Molecular mechanisms of photoreceptor-mediated light signaling in plant cells. This diagram illustrates the signaling pathway used to send low and high light signals and responses in plants. Photoreceptors; Phot1 and Phot2, in the cell membrane; these molecules detect the intensity of light. Low light response protein Jac1 is activated by Phot1 and Phot2 in the cytoplasm, and they then transmit a signal to the nucleus. High light response Phot2 is in direct contact with the chloroplast protein CHUP1 and blue light’s CP FKF1 and degrades FKF1. Intracellular interaction with blue light affects cp-actin, which causes signal accumulation and subsequent reaction in the golgi bodies and chloroplasts. The extensive interconnected network ensures that plants can alter their growth and development depending on the light.

Although it is essential for the photoprotection of chloroplasts from high light, the escape machinery remains poorly characterized. Photoreceptors driving chloroplast relocation especially phototropins (PHOTs), principally PHOT1 and/or PHOT2, work as the key photoreceptor in recent studies. While the low light accrual response is invoked by both, PHOT2 appears to be significantly more important for high light avoidance (**Figure 2**). Many studies have reported that chloroplast actin filaments or cp-actin play a crucial role as the mechanical force generator for the movement of chloroplasts and their quantity is related to the speed of mobility (Wada, 2013). These cp-actin filaments induce the displacement of chloroplasts so that smooth rotation without rotational motion is made possible (Kadota et al., 2009). The pathways through which light is absorbed and utilized by the plant are illustrated, highlighting how each chloroplast in *Capillus veneris* (T-1 ~ T-x) or *Arabidopsis thaliana* is capable of changing its orientation in response to light intensity, optimizing light absorption and minimizing photodamage (Tsuboi et al., 2009). There are several proteins identified as key regulators of chloroplast positioning, including Chloroplast Unusual Positioning 1 (CHUP1), Plastid Movement Impaired 1 (PMI1), Kinesin-like protein for Actin-based Chloroplast Movement (KAC), and THRUMIN1. By interacting with these elements, chloroplasts are anchored to the cell and can move around, with CHUP1 facilitating the polymerization and association of cp-actin filaments with the plasma membrane through THRUM1 (Ishishita et al., 2020).

## 5 Anthocyanin biosynthesis and its role in light stress acclimation

**Figure 3:**
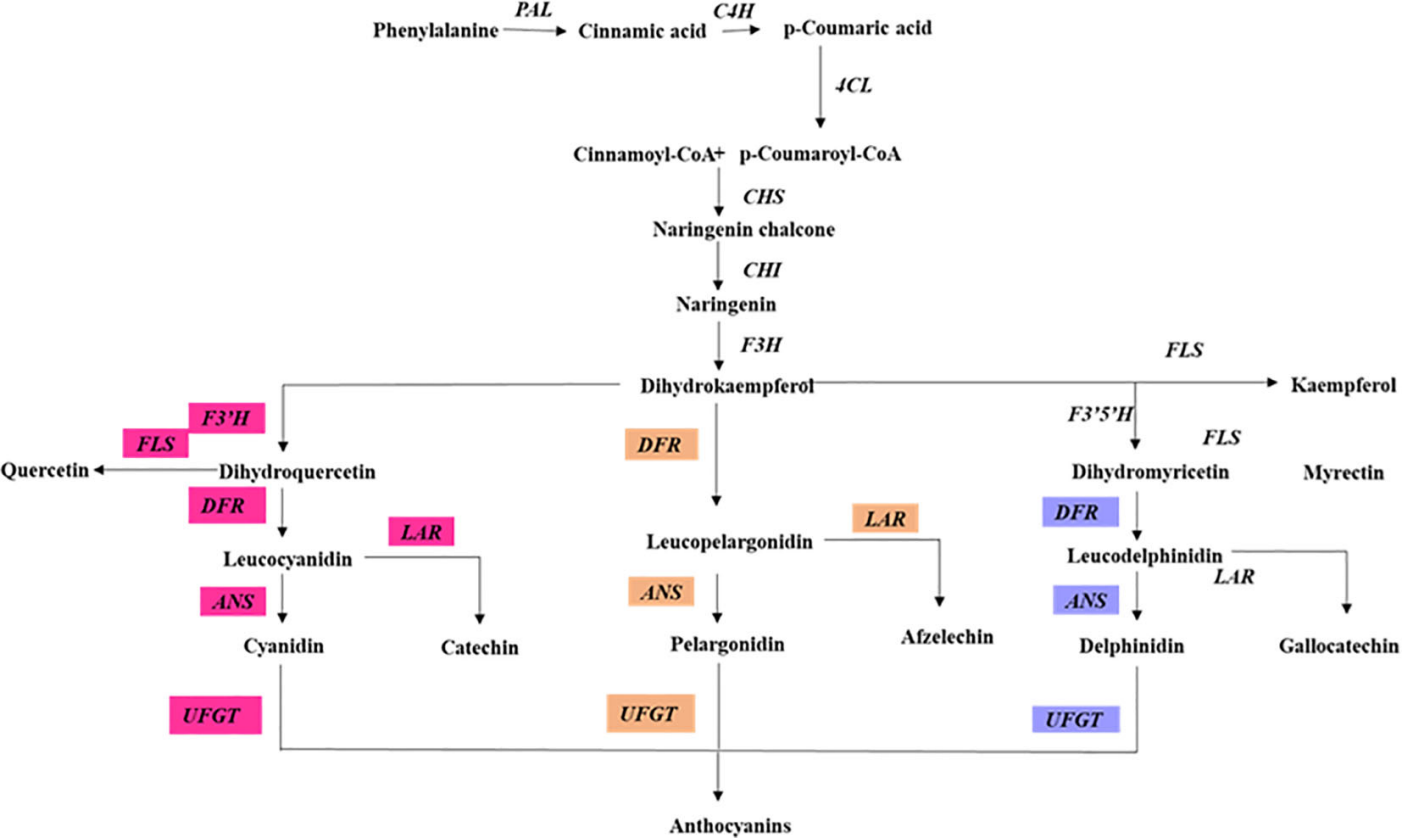
Anthocyanin biosynthesis pathway in plants. This diagram illustrates the enzymatic steps involved in the biosynthesis of anthocyanin. Highlighting the key enzymes and their respective positions in the pathway. PAL, phenylalanine ammonia-lyase; C4H, cinnamate 4-hydroxylase; 4CL, 4-coumarate CoA ligase; CHS, chalcone synthase; CHI, chalcone isomerase; F3H, flavanone 3-hydroxylase; F3′H, flavonoid 3 hydroxylase; F3′5′H, flavonoid 3,5 hydroxylase; FLS, flavonol synthase; DFR, dihydroflavonol 4-reductase; ANS, anthocyanidin synthase; UFGT, UDP-galactose flavonoid 3-O-galactosyltransferase; The pathway is divided into different branches, each leading to the synthesis of anthocyanin biosynthesis.

Anthocyanins, water-soluble pigments in plants (**Figure 3**), play a pivotal role in mitigating photoinhibition and photo-oxidative damage under high light intensity (Zheng et al., 2021; Fu et al., 2023). Throughout their evolutionary history, plants have evolved various mechanisms to adapt to and mitigate high light conditions. Among these, the synthesis and accumulation of non-photosynthetic pigments, particularly anthocyanins, stand out as a crucial adaptation, enabling plants to regulate the absorption of light energy more effectively (Albert et al., 2009). Localized typically within vacuolar epidermis cells, anthocyanins act as front-line protection against high light stress by giving specific photoprotective effects (Hughes et al., 2005). In higher plants, anthocyanins begin with their biosynthesis as part of the general flavonoid framework (Shi and Xie, 2014; Tohge et al., 2017). This intricate chain of reactions is orchestrated by structural and regulatory genes (Ahmed et al., 2015; Feng et al., 2013; Shin et al., 2015; Xie et al., 2014). Anthocyanins function as both an antioxidant ROS sponge and a light absorber, which emphasizes their value in plant survival strategies. In high concentrations, they disassemble thylakoid membranes; in other words, evident from the leaves of poinsettias, they resist ROS formation and protect PSII complexes from being damaged (Moustaka et al., 2020).

Besides, anthocyanins form an indispensable part of sunscreen (in terms of radiation filtering) for preserving improved photoprotective systems in plants. This is especially obvious in situations where more plants can harvest the green light using anthocyanins under high white light than they possibly to photosynthesis with red or blue (Terashima et al., 2009). Analysis of an even more complex anthocyanin regulatory gene in *Arabidopsis thaliana* suggested that their accumulation is induced, not during darkness as is often considered (Gonzalez et al., 2008 *)* but specifically by high light or increased temperature – a targeted response to environmental stress factors involving both bHLH and MYB-R2R3-type transcription factor. For example, the transcription factor HY5 in *Arabidopsis thaliana* plays a key role. it binds to the MYB75 (PAP1) promoter and induces an increase of anthocyanin production under high light by activating its expression (Shin et al., 2013; Jiang et al., 2024). This evolutionary trend of anthocyanins as a defense system showcases the flora to perceivably superior environment challenges. Therefore, protective agents like anthocyanins have a crucial mechanism for efficiently lowering ROS, scavenging over-exposed radiation, and tuning light absorbance to reduce most photoinhibition and photo-damage related risks significantly. In addition, they do more than protective mechanisms and enhance the visual appeal and overall resilience of plants. Anthocyanins are not simply colorants, here we explore the intriguing multitude of roles these pigments can play in plant stress responses and adaptive strategies.

## 6 Responses of photosynthetic apparatuses to light stress

Under conditions of intense light exposure, PSII within plants can become rapidly deactivated, leading to a swift reduction in photosynthetic effectiveness, a process referred to as photoinhibition (Roach and Krieger-Liszkay, 2014). To counteract the detrimental effects of excessive light, plants employ mechanisms like photochemical and non-photochemical quenching to dissipate the surplus energy absorbed by PSII (Szymańska et al., 2017). This adaptation is part of a broader spectrum of responses that include state transitions, which are adjustments in the plants’ light energy processing systems in response to changing light conditions (Haldrup et al., 2001). Different plants have evolved various strategies to manage and mitigate high-light stress. For instance, phototropins are crucial for initiating movements in chloroplasts that help them avoid excess light, thereby protecting the photosynthetic machinery (Jarillo et al., 2001). In *Arabidopsis thaliana*, cryptochromes play a significant role in triggering the activation of numerous genes that respond to high light conditions, showcasing a complex regulatory network in response to light stress (Kleine et al., 2007). Furthermore, UVR8, a specific receptor sensitive to UV-B light, is instrumental in activating gene expression pathways that not only mitigate the effects of UV-B radiation but also assist in DNA repair and reducing damage from oxidative stress, demonstrating the sophisticated defense mechanisms plants have developed to cope with varying light intensities (Brown and Jenkins, 2008; Favory et al., 2009).

Under low light conditions, the photosynthetic mechanism in plants undergoes significant adjustments through the modification of enzyme levels involved in the carbon-reduction cycle (Wang et al., 2013), alterations in electron transport components (Yang et al., 2018b), adjustments in protein concentrations (Li et al., 2014b), and changes in the amounts of light-harvesting pigments (Wherley et al., 2005). These alterations contribute to a reduction in photosynthetic rates, as observed across various plant species (Sarijeva et al., 2007; Jiang et al., 2011; Li et al., 2014a; Yang et al., 2018a; Muneer et al., 2014). This decline in photosynthetic performance is typically associated with reduced electron flow through PSII (Yao et al., 2017). Shade-adapted plants exhibit reduced quantum yields (Fv/Fm), effective quantum yields of the photosystems (PS), photochemical quenching (qP), and electron transport rates (ETR) (Hussain et al., 2019). The stress from the shade, particularly during the vegetative growth stage, impedes photosynthesis by obstructing the electron transport from PSII to (PSI, leading to a decline in the electron transport rate, a decrease in ATP synthesis, and diminished activity of the enzyme Rubisco (Yao et al., 2017; Huang et al., 2018). In response to these light condition alterations, plants make molecular-level adjustments to their photosynthetic machinery, specifically by modulating the sizes of the PSI and PSII antenna complexes relative to light intensity; plants grown under low light conditions develop larger antennas compared to those grown under high light conditions (Anderson et al., 1995). This adaptive strategy enables plants to optimize their photosynthetic efficiency under varying light environments.

### 6.1 Chloroplastic ROS

**Figure 4:**
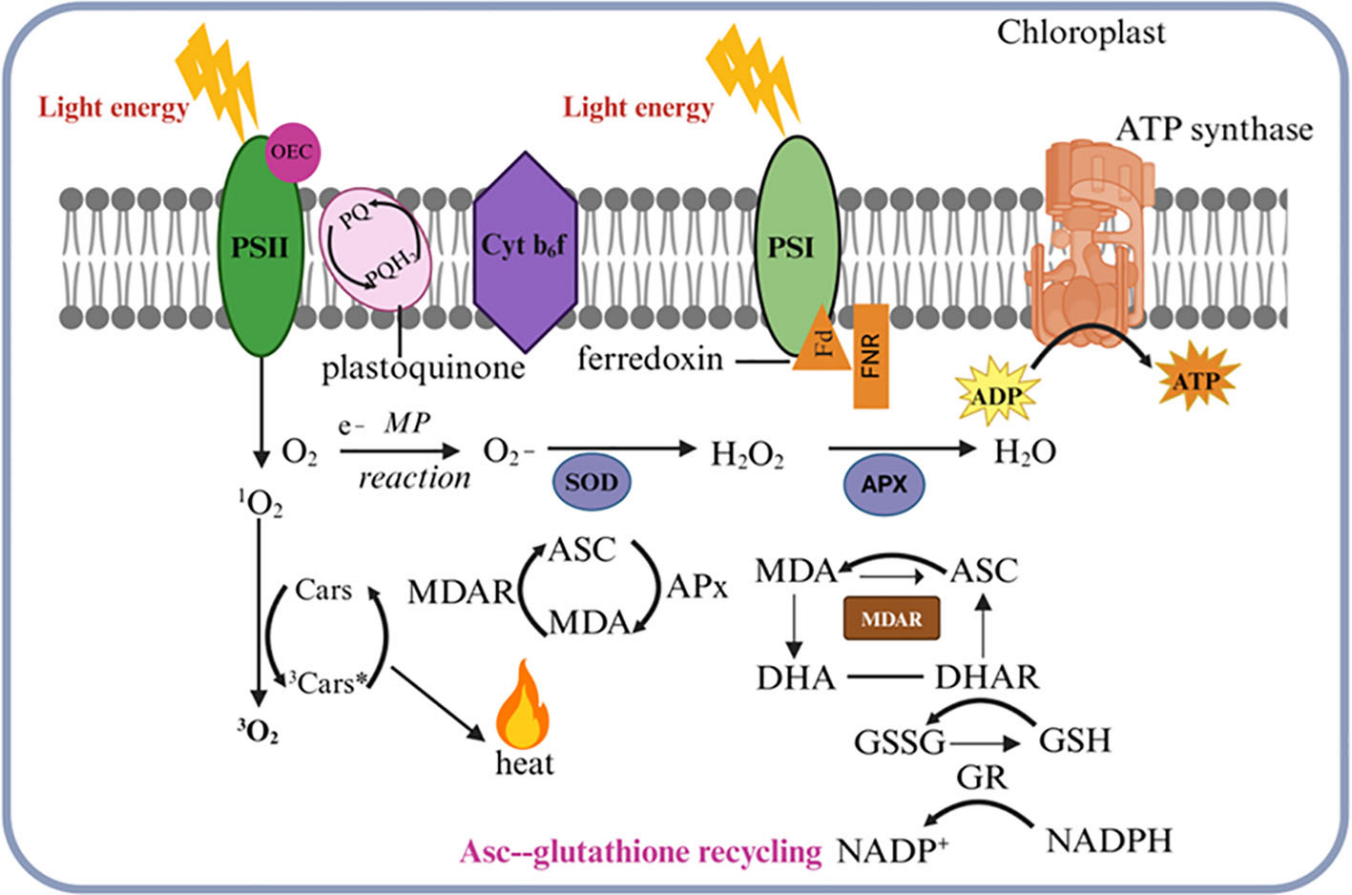
Reactive oxygen species (ROS) detoxification in the chloroplast. Singlet oxygen (1O_2_) is generated when oxygen interacts with the triplet-excited state of chlorophyll in photosystem II (PSII). The excitation energy is transferred to carotenoids (Cars), resulting in the production of triplet molecular oxygen (3O_2_) and excited triplet carotenoids (3Car*). The 3Car* then dissipates this energy as heat while reverting to its ground state. In photosystem I (PSI), oxygen photoreduction can produce superoxide (O_2_), which is neutralized by superoxide dismutase (SOD) to form hydrogen peroxide (H_2_O_2_). This H_2_O_2_ is then reduced to water H_2_O by ascorbate peroxidase (APX) using ascorbate (Asc), leading to elevated levels of mono-dehydroascorbate (MDA) and dehydroascorbate (DHA). MDA and DHA are reduced by monodehydroascorbate reductase (MDAR) and dehydroascorbate reductase (DHAR), respectively. These compounds are subsequently reduced by monodehydroascorbate reductase (MDAR) and dehydroascorbate reductase (DHAR), respectively. Additionally, during the ascorbate-glutathione recycling process, NADPH-dependent glutathione reductase (GR) facilitates the reduction of oxidized glutathione (GSSG) back to its reduced form, glutathione (GSH). This diagram illustrates how light absorption, electron transport, and antioxidant defense mechanisms are integrated within the chloroplast.

Light stress results in an overproduction of chloroplastic ROS that is associated with signaling and increased oxidative damage. ROS are now recognized as key regulatory molecules in plants and participate in the early signaling transduction caused by all types of stress despite the earlier assumption that ROS are harmful products of aerobic metabolism (Exposito-Rodriguez et al., 2017; Irfan et al., 2017). To regulate the production of chloroplastic ROS light energy must be captured and sent to photosynthetic devices. The main contributors to chloroplastic ROS are the PSII reaction center, the electron transport chains of PSI, and the light-harvesting complex II (LHCII) (Nelson and Ben-Shem, 2004). Their production increases during exposure to light stress, especially if the concentration of CO_2_ declines and ATP production is reduced (Takahashi and Badger, 2011). One-electron oxygen photoreduction can produce hydrogen peroxide (H_2_O_2_) and other moderately reactive peroxides, particularly at PSI. Ferredoxin (Fd)-mediated electron transfer from PSI results in the creation of superoxide during the Mehler-Peroxidase (MP) reaction (Li et al., 2009). The chloroplast’s ROS are neutralized by a sophisticated antioxidant network, which reduces photodamage (Bassi and Dall’osto, 2021). Accumulating chloroplastic ROS depends on balancing generating and detoxifying ROS via the scavenging network. Enzymatic antioxidants and ROS scavengers are included in the detoxification systems. 3O_2_ and 3Car* formation results from the excitation energy moving from ROS to carotenoids (Cars). The exciton energy is safely converted into heat as 3Car* decays into the ground state in a gradual process. Ascorbate peroxidase (APX), another potent ROS-scavenging enzyme, efficiently uses ascorbate (ASC) to convert the highly reactive H_2_O_2_ molecules into water molecules. The scavenging of these harmful free radicals by APX results in increased levels of the oxidized forms dehydroascorbate (DHA) and mono-dehydroascorbate (MDA). High accumulations of both MDA and DHA are promptly reduced by the coordinated actions of MDA reductase (MDAR), glutathione reductase (GR), and DHAR, working in concert. Moreover, during this critical ascorbate-glutathione redox recycling reaction, GR significantly accelerates the reduction of the oxidized glutathione (GSSG) to its reduced form glutathione (GSH) to maintain a higher ratio of beneficial GSH relative to GSSG within the chloroplast stroma (**Figure 4**) Gururani et al., 2015. Besides ROS scavenging, chloroplast positioning, stomatal regulation, and increased anthocyanin production, chloroplasts also sense light stress and transmit the signal to the nucleus leading to changes in gene expression that regulate the stress response. This regulation involves retrograde signaling, where chloroplast signals communicate with the nucleus to adjust gene expression. A number of signaling molecules activate nuclear gene expression in response to high light stress, including Mg-Protoporphyrin IX (Mg-ProtoIX), heme, and ROS (Woodson and Chory, 2008). During high light stress, transcription factors play an important role in modulating gene expression. HY5 (elongated hypocotyl 5) acts as a central regulator, binding to light-responsive elements in target gene promoters, and enhancing the expression of genes involved in photoprotection, such as chlorophyll-binding proteins and ROS-detoxifying enzymes (Li et al., 2023). Furthermore, bZIP transcription factors (such as HY5 and HYH) regulate gene networks that influence photomorphogenesis and oxidative stress responses (Dröge-Laser et al., 2018). Under high light conditions, phytochrome-interacting factors (PIFs) are negatively regulated to prevent excessive elongation and reduce photodamage (Leivar and Monte, 2014). As a result of fluctuating light conditions, salt tolerance (STO) protein and its homologs help regulate gene expression (Indorf et al., 2007). Under high light conditions, plants fine-tune their stress responses by coordinating these regulatory pathways at the genetic level.

### 6.2 Response of chloroplast to low light and high light stress

Plants are exposed to various stresses under natural environmental conditions. Plants need a suitable amount of light and heat to grow healthily, but when either of these conditions is extreme, it puts plants under stress. The quantity and quality of sunlight vary according to the time of day and the weather. Under every changing environmental condition, the chloroplast often moves in the cell to capture light energy efficiently. Instead, in the chloroplasts, light-harvesting Chl-protein complexes move along the thylakoid membranes to absorb light energy (Yamamoto, 2016). Conversely, when the chloroplasts or the Chl-protein complexes receive extreme light from the sun, they avoid or tolerate the light stress using short- and long-term strategies. Under natural environmental conditions, low or high-temperature pressure is superimposed on light stress, which makes the situation more complex. The primary focus is on the mechanisms that higher plant chloroplasts use to avoid and tolerate light and heat stresses at the thylakoid level, with special attention given to the role of thylakoid membrane fluidity, which is crucial for the effective functioning of these avoidance and tolerance mechanisms. Low light stress seriously affected the chloroplasts in tall fescue, causing morphological cell disruption, chloroplast number reduction, disordered thylakoid grana lamellae, and chloroplast membrane damage (Long et al., 2022).

Studies have shown that low light stress disrupts chloroplast structure, lowers Rubisco enzyme activity, reduces CO_2_ assimilation, and ultimately limits plant growth. In contrast, high light stress damages the photosynthetic apparatus, particularly PSII and Rubisco (Sharma et al., 2020). As a result of high light exposure, ROS accumulate, causing oxidative damage to chloroplast membranes and proteins, further reducing photosynthetic efficiency. As low light stress restricts light capture, high light stress inhibits photosynthesis through photoinhibition and oxidative damage (Lu et al., 2019). Ultimately, both stresses reduce plant growth, but through different mechanisms (Huylenbroeck and Bockstaele, 1999).

### 6.3 Thermal energy dissipation

Plants dissipate high light energy as heat to prevent ROS damage, which is among the most efficient and rapid photoprotective mechanisms (Foyer, 2018). When plants are exposed to extremely high light intensities that exceed their energy needs or their ability to dissipate the high light, a range of high light stress responses can occur (Ruban and Murchie, 2012). Typically, non-photochemical quenching (NPQ) is regarded as one of the quickest mechanisms for modulating light harvesting, playing a crucial role in protecting PSII reaction centers from photodamage, which can lead to photoinhibition. NPQ causes dissipation of excessive excitation energy in the form of heat (Ruban and Murchie, 2012). For *in vivo* NPQ the minimal requirements to sustain NPQ in living organisms are as follows: high-energy quenching (qE) needs stable state transition establishment using DPH 70 Figsand Sbs. Based on their recovery kinetics, we have found several activities that arise from NPQ. This state transition process leads to the reduction in LHCII between PSII and PSI, providing redistribution of photo-energy absorption from both photosystems (Bellafiore et al., 2005; Foyer, 2018). Processes that decrease PSII quantum yield in the light are collectively termed photoinhibition (qI). A rise in net value may be the result of several properties with slow relaxation kinetics, including PSII photoinactivation (photoinhibition) and unidentified modes of continuous thermal dissipation (Demmig and Björkman, 1987). Heat dissipation (qE) relies on a low pH within the thylakoid lumen and involves the PsbS subunit of LHCII, as well as the conversion of violaxanthin to antheraxanthin and zeaxanthin through the xanthophyll cycle (Mi et al., 2000; Kromdijk et al., 2016).

## 7 Photosystem

Photosystems are the functional units for photosynthesis, defined by a particular pigment organization and association patterns, whose work is the absorption and transfer of light energy, which implies the transfer of electrons. Physically, photosystems are found in thylakoid membranes.

### 7.1 Photosystem-I

light energy is as crucial for photosynthetic electron transport but can be harmful to the system when provided more than sinks within chloroplasts capable of absorbing electrons. In this way, electrons or excitations are extracted from O_2_ to ROS which oxidizes proteins, lipids, and metabolites but also generates signaling compounds as indicated below. These photo-oxidative conditions arise from alterations in environmental conditions. This damage is manifested as a form of “photoinhibition” (Gururani et al., 2015). Which has been defined as the inactivation of one, or even both photosystems. This inhibition is considered to be associated with the reduction of photosynthetic capacity and in return limits plant growth and crop yield (Takahashi and Murata, 2008; Kato et al., 2012; Simkin et al., 2017), which contrasts with damage by excessive light to PSII that often results from photoinhibition (Tyystjärvi and Aro, 1996). Photoinhibition of PSI is prevented by several mechanisms.

On the other hand, PSI photoinhibition results from the over-reduction of PSI relative to the conventional stromal acceptors and the subsequent reduction of O_2_ as an alternative electron acceptor. O_2_ reduction occurs both at the PSI acceptor side and the pheQ A1 site. In nature, the product is the radical superoxide (O_2_•−) which in turn dismutates to H_2_O_2_and oxygen O_2_ (Takagi et al., 2016; Kozuleva et al., 2021). The PSI photoinhibition is a result of the reaction of the H_2_O_2_ and the FeS clusters to yield hydroxyl radicals and at the same time deactivate PSI electron transport (Sonoike et al., 1997; Sonoike, 2011). The damage to protein subunits also plays a role in inhibiting the PSH through the activities of O_2_ and singlet oxygen (1O_2_), which are produced from the reactions of triplets P700 and P680 (Takagi et al., 2016). The mechanism of production of ROS and the photoinactivation of the PSI is also not inferred. In the current world, photoinhibition of the PSI is a result of environmental stressing factors like low temperatures, drought, and high salinities. All these factors are known to limit the assimilation of CO_2_ (Munekage et al., 2008; Takahashi and Murata, 2008). There is also photoinhibition when the unregulated electron flow is more than the PSI acceptor side capacity (Munekage et al., 2002; Suorsa et al., 2012; Tiwari et al., 2016; Kanazawa et al., 2017; Lima-Melo et al., 2019). Frequent fluctuations in light intensity also contribute to the photoinhibition of PSI (Tikkanen and Grebe, 2018; Kono and Terashima, 2016). The evidence available highlights that in these environments there is a higher rate of PSI photoinhibition in red and blue light. These lights are predominantly absorbed by the PSII through the P680 reaction site. The white and green light has less effect on PSI photoinhibition (Oguchi et al., 2021). The PSI is inhibited by the photosynthetic electron transport especially when the chloroplast’s sink capacity is in excess.

PSI inactivation is energy-dependent (as mentioned, that being simply the amount of “energy imbalance” between donor and acceptor available). Therefore, the photoinhibition avoidance mechanisms of PSI are different from two sides (Shimakawa and Miyake, 2018). These mechanisms comprise PSII reaction center inactivation, light absorption energy dissipation into heat, and electron flow limitation through/around cyt b6f (Tikkanen and Aro, 2014). Under this photoprotective scenario, PSII-independent barriers to electron flow serve to alleviate PSI acceptor side reduction and drive oxygen O_2_ photo-mitigation. Excess energy absorbed by PSII is a primary trigger for the generation of 1O_2_ in the PSII reaction center leading to damage to core D1 protein and arrest of any further photosynthetic activity until replacement of damaged D1 (Nixon et al., 2010). The temporary inactivation of PSII may assist the liberation of excitation constraints on other PSI molecules when PSII is alleviated from excessive excitations and that could prevent over-reduction of PSI (Tikkanen and Aro, 2014; Huang et al., 2016). The protonation of PsbS and the xanthophyll cycle activation by acidification in the thylakoid lumen leads to a NPQ process that dissipates over-energization from LHCII (Ruban and Murchie, 2012). Aside from quenching, NPQ also protects PSI from excess excitation energy by preventing heat loss via the LHCII antenna pool associated with PSII (Tikkanen and Grebe, 2018; Hepworth et al., 2021), and indirectly through reductions in downstream electron pressure due to reduced PSII activity (Han et al., 2010; Sonoike, 2011; Chaux et al., 2015). Furthermore, the acidification of the lumen as a result of NPQ actuation followed by the establishment of pH gradients across the thylakoid membrane slow PCET through the Cyt B6F complex (Tikhonov, 2014). Moreover, this regulation mechanism (photosynthetic control) protects PSI from overreduction on both the donor and acceptor side in times of rapid light increases. PGR5 is required for NPQ and, concomitantly photosynthetic control so that plants deficient in the PGR5 protein undergo inhibition of PSI to a much larger degree when subjected to increases in light intensity (Munekage et al., 2002; Suorsa et al., 2012; Tiwari et al., 2016; Gollan et al., 2017; Takagi and Miyake, 2018; Lima-Melo et al., 2019). The pgr5 mutation does not disable NPQ and the lack of PSI photoinhibition (Gollan et al., 2017; Rantala et al., 2020), shows that the photosynthetic state is a crucial factor in determining how quickly cells can induce per-PSI under dynamically changing environments (Padmasree et al., 2002).

The PSI photoprotection mechanism also depends on the acceptor side capacity, and an enhanced electron transport toward efficient stroma sinks might reduce or protect against this deficit in PSI (Padmasree et al., 2002; Alric and Johnson, 2017; Wada et al., 2018; Yamamoto and Shikanai, 2019). Flavodiiron (FLV) proteins, which can oxidize electron carriers downstream of PSI as found in cyanobacteria and algae are missing from angiosperms; even though they can be detected in a number of lower-order land plants including gymnosperms (Zhang et al., 2009; Gerotto et al., 2016; Ilík et al., 2017). Recent studies have indicated the re-introduction of FLV proteins to angiosperm chloroplasts which has strongly demonstrated value in increasing stromal sink strength and effective PSI protection from over-reduction that likely involves a contribution to improved electron transport capability, lumen protonation stimuli for NPQ induction/development and associated photosynthetic control (Wada et al., 2018; Yamamoto and Shikanai, 2019). The major natural electron sink in the chloroplast is the reduction of CO_2_ into sugars by CBB cycle activity. Cyclic thylakoid influences improved protection of PSI during fluctuating light by elevated CO_2_ levels (Tan et al., 2021).

### 7.2 Photosystem-II

Photosynthesis consumes the coordinated function of several large membrane complexes: PSII, the Cytochrome b6f complex, PSI, and ATP synthase. PSII is a pigment-protein complex of the integral membrane, with many subunits splitting water into O_2_, protons, and electrons in the course of photosynthesis (Nickelsen and Rengstl, 2013). The *de novo* assembly and reassembly of PSII in cyanobacteria and vascular plants are extremely conservative. There are three stages in the process of *de novo* assembly of PSII, 1. Assembly of the intermediate the reaction center subcomplex. 2. Assembly of the monomeric PSII-complex and its dimerization. 3. Assembly of the PSII-LHCII supercomplex. The formation of the reaction center is achieved by the combination of two subcomplexes, such as pD1-PsbI, and D2-Cyt b559 (Boehm et al., 2012). The low-molecular-mass subunits of PSII, including PsbH, PsbM, PsbT, and PsbR, form the RC47 complex, which is associated with the reaction center subcomplex and the CP47 complex (Boehm et al., 2012). In the next stage, CP43 and low molecular mass PsbK sequentially attach to RC43, leading to the formation of a PSII monomer that does not capture oxygen, called an oxygen-evolving complex. LMM subunit dimerizes the PSII core monomer and associates it with the peripheral antennae to form a PSII-LHCII supercomplex (Bricker et al., 2012). Accordingly, when PSII assembles, many transiently interacting facilitators are available for different intermediate subcomplexes that are not parts of the functional PSII (Nixon et al., 2010). In addition, the conversion of precursor D1 into mature D1 of the PSII RC complex is involved in the formation of the complex (Che et al., 2013). However, it should be noted that the assembly of the PSII core proteins was considerably affected following the absence of the C-terminal processing of pD1, with the greatest effects observed in the subsequent assembly of PSII core proteins, especially for CP4 (Shi et al., 2021b, Shi et al., 2021a). The identified PSII core proteins also interact with the protein known as photosynthesis wasteland mutant 68, and disruption of PAM68 function inhibits the conversion of RC complexes to PSII complexes (Armbruster et al., 2010). CpSRP43 is an ATP-independent chaperone, and it can rescue Sung1 aggregation *in vitro* as well as similar to the chloroplast signal recognition particles from agarose including GluTR, CHLH, and GUN4 (Ji et al., 2021). Additionally, cpSRP43 binds to cpSRP54 to facilitate the transfer of newly imported LHCPs onto the ALB3 translocase, releasing them from cpSRP43 and integrating them into the thylakoid membrane (Moore et al., 2000). In order for PSII to function properly, each assembly factor must act in a highly coordinated manner. In high light stress, however, high light energy can inhibit PSII, resulting in a reduction in electron transfer efficiency. As a result of this photodamage, ROS accumulate, which can damage the reaction center and other proteins. As a result of low light stress, PSI and PSII are unable to absorb enough light for photosynthesis, resulting in reduced electron flow and reduced photosynthetic efficiency.

### 7.3 Photodamage of PSII

When the amount of light energy absorbed surpasses the photosynthetic electron transfer chain’s capacity, the photosynthetic machinery faces significant risks of oxidative damage (Gururani et al., 2015). Extensive research has been conducted on the occurrence of PSII photoinhibition under high-light conditions, revealing that PSII-LHCII supercomplexes are particularly vulnerable to such damage. It has been demonstrated that under intense light stress, PSII primarily suffers damage due to the direct absorption of light by the oxygen-evolving complex (OEC), leading to the release of manganese ions (Mn^2+^) and the disruption of Mn^2+^ clusters. It is suggested that, without a functional OEC, the excess light energy is absorbed by chlorophyll and carotenoids, which subsequently damages the PSII reaction center (Takahashi et al., 2010). The extent of PSII photoinhibition under high light stress is determined by the balance between damage and repair (Gollan and Aro, 2020). Additionally, high light stress negatively impacts the components of the photosynthetic electron transport chain (PETC).

In addition, PSII and PSI trigger the production of ROS, which further damages the chloroplasts by oxidative stress. Additionally, ROS suppresses D1 biosynthesis, inhibiting PSII repair. Further, high levels of UV-B radiation (280 nm–315 nm) are associated with high intensity of sunlight. The ozone layer has been able to remove a large portion of the UV-B component of sunlight from the stratosphere. However, the remaining UV-B component of sunlight that reaches the earth’s surface can still be harmful to all living organisms (Demarsy et al., 2018). DNA and proteins are macromolecules that UV-B can damage. Plants also accumulate harmful ROS due to UV-B damage. Moreover, UV-B can destroy the Mn clusters in OEC, causing photoinhibition and serious damage to PSII (Takahashi et al., 2010; Takahashi and Badger, 2011).

### 7.4 Photorepair of PSII

When PSII is impaired as a consequence of light stresses, specific protection and repair mechanisms are in place to restore its activity. The central process in the PSII repair cycle is degradation and restoration of the D1 protein subunit within PSII repairing damage to the reaction center core complex, thus preventing inhibition of photosynthetic electron transport (Didaran et al., 2024). A photorepair mechanism occurs when PSII and LHCII are phosphorylated under high light stress, enhancing the photosynthetic apparatus’ recovery from damage. On the other hand, the phosphorylation activity of LHCII appears to be performed by state transition 7 (STN7) (Pesaresi et al., 2011), while that for PSII core proteins is mostly carried out by a kinase called state transition 8 (STN8). STN8 has been shown to phosphorylate core complex proteins of PSII as well (Pesaresi et al., 2011; Bellafiore et al., 2005). Additionally, PSII core monomers migrate from the grana stacks to stroma-exposed membranes where CPP43 and OEC complexes disassemble producing a damaged D1 protein-containing RC47* complex after the impairment of D1 protein and severe, a new synthesized D1 assembles into RC47. At this point, the released CP43 and OEC peptides rebind to RC47, forming a new PSII monomer. These restored PSII monomers are subsequently transported to the Grana core for dimerization and binding with peripheral antennae in order to establish functional PSII-LHCII super complexes (Theis and Schroda, 2016).

### 7.5 Sequence of PSII assembly

**Figure 5:**
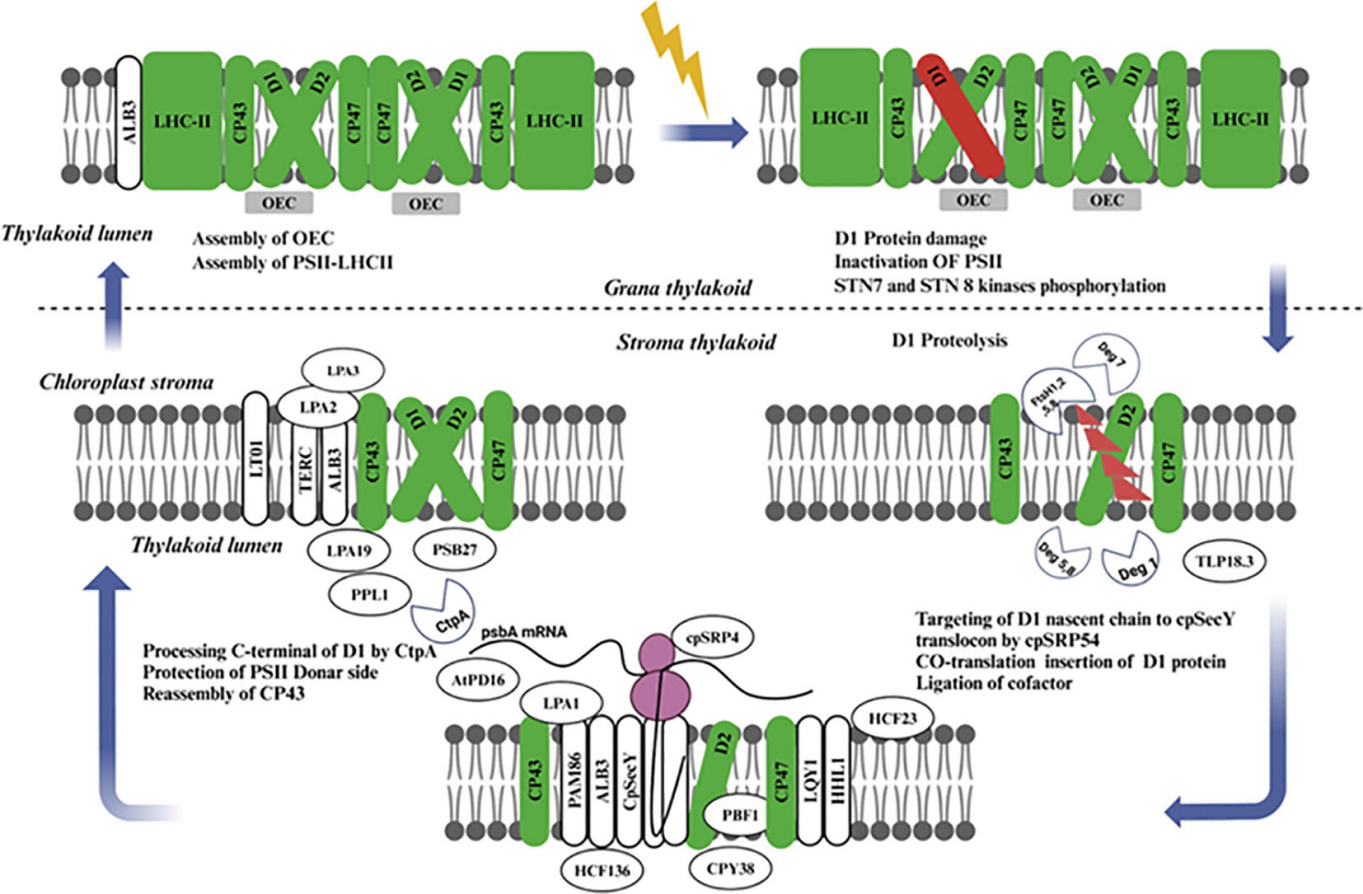
Schematic representation of the coordinated processes involved in the damage recognition, degradation, and repair of PSII, emphasizing the essential role of protein turnover for photosynthetic efficiency and stability under fluctuating light conditions. The interplay between assembly factors, chaperones, and proteases ensures the continuous function of PSII, maintaining optimal photosynthetic performance. PSII, Photosystem II; LHC-II, Light Harvesting Complex II proteins (green); OEC, Oxygen Evolving Complex (gray); Light-induced damage of D1 protein (red), cpSRP54, Chloroplast Signal Recognition Particle 54; cpSecY, Chloroplast SecY; STN, STN Kinase; LPA, Low PSII Accumulation; AtPD, Arabidopsis Thylakoid Protease.

In recent years, numerous research and review papers have been published on the biosynthesis, assembly, and repair of PSII. These studies have significantly advanced our understanding, particularly with the identification and characterization of PSII auxiliary proteins and the regulatory mechanisms involved in the PSII assembly and repair cycle (**Figure 5**) (Mulo et al., 2008; Komenda et al., 2012; Pagliano et al., 2013a). Detailed insights into the assembly intermediates of newly synthesized PSII complexes and those undergoing repair have been gained through stepwise pulse-chase labeling experiments combined with the analysis of protein complex compositions via native gels (Järvi et al., 2015). Further understanding of PSII synthesis and repair mechanisms has come from studying mutant plants that lack specific structural subunits or auxiliary proteins of PSII, as well as experiments examining the transition from etioplasts to chloroplasts (Rolo et al., 2024). While the assembly process of PSII in higher plants, algae, and cyanobacteria is quite similar, with several auxiliary factors being evolutionarily conserved, there are notable differences (Liu et al., 2019). Therefore, this discussion will focus on PSII repair in plant chloroplasts.

Some components of the PSII repair cycle and *de novo* biogenesis have common features. But, in the PSII repair cycle, only reaction center protein D1 and occasionally the D2, CP43, and PsbH subunits, are replaced (Nelson et al., 2014; Järvi et al., 2015), except for these subunits, nearly all other component of the complex are recycled. The repair cycle starts with the monomerization of phosphorylated dimeric PSII complex in grana stacks and is continued by dephosphorylation of D1, D2 core proteins and CP43 most probably while passing through only loosely stacked non-appressed stroma-exposed thylakoids. Next, there is partial disassembly of the PSII core complex leading to the release of CP43 inner antenna protein, OEC proteins, and other unknown low molecular mass subunits followed by proteolytic degradation from D1. Here translation of the PSII α and β subunits facilitates assembly with Cytb559, while D2 is synthesized as a core protein (Kanervo et al., 2008; Suorsa et al., 2004). This smallest subcomplex of PSII is composed of Cyt b559, D1, and PsbI, but notably lacks D2 (Rokka et al., 2005) Other low-molecular-mass (LMM) subunits may also be present in this complex, but their precise roles and compositions remain unclear. A further investigation of the functions of these additional proteins is necessary in the future.

Light regulates psbA mRNA translation initiation and the chloroplast signal recognition particle cpSRP54 directs nascent D1 chain present on translating ribosome to insert co-translationally through thylakoid membrane (Nilsson et al., 1999), where, D1 protein is inserted into PSII core subcomplex via the cpSecY translocation channel (Zhang et al., 2001). A ribosome pauses on psbA mRNA while the newly translated D1 protein elongates, and this is thought to enable nascent chains its binding cofactors, such as chlorophylls long before polypeptide release from the ribosomes, the D1 protein has 5 transmembrane helices with N-terminus facing stroma in thylakoid membrane (Zhang and Aro, 2002) with weak interaction between the first two transmembrane helices of nascent D1 chain and the protein. After translation, which only comprises four transmembrane segments in the case of PSII (D1), a strong association becomes apparent (Knoppová et al., 2022). The assembly of the inner antenna protein CP47 follows, along with the assembly of low molecular mass proteins PsbH, PsbL, PsbM, PsbTc, PsbR, and possibly PsbJ (van Bezouwen et al., 2017); Pagliano et al., 2013b). During *de novo* assembly of PSII, it is conceivable that some of these proteins remain attached to the PSII core throughout the repair process rather than being released. The low molecular mass subunits of PSII play a crucial role in stabilizing the CP43-less PSII core complex, which represents the smallest visible PSII repair cycle intermediate in silver-stained gels. The C-terminal processing of the D1 protein (Dobáková et al., 2009) likely occurs before the assembly of CP43, PsbK, and PsbO proteins. The subsequent steps involve the assembly of PsbW and PsbZ, which control the interaction of the PSII core with LHCII (García-Cerdán et al., 2011), followed by PSII dimerization and the formation of PSII-LHCII supercomplexes in the appressed grana thylakoids.

## 8 Conclusion and perspectives

Plants in their natural habitats face various stresses, including light stress, which can reduce photosynthetic efficiency and vary from brief periods to several months. Effective protection strategies are crucial for their growth, survival, and reproduction. Plants have developed multiple defense mechanisms, such as regulating redox-active molecules, scavenging ROS, decreasing light absorption, and modifying their transcriptome and metabolome. Recent research has shed light on how plants balance efficient light harvesting with protection. They trigger scavenging ROS, adapting chloroplast and stomatal movement as well, and finally synthesizing anthocyanins for downstream machinery to capture more energy while preventing damage. Number-wise, indispensable help comes from plant hormones or physical and small molecules in reducing light stress. Light stress underscores a serious problem for plants, resulting in considerable negative effects such as the inactivation of PSII also termed photoinhibition. In response to this, plants call in to play the two repairing pathways for PSII and NPQ to mitigate any further damages.

Furthermore, it enhances the expression of chloroplast proteins that maintain and repair photosystem, preserving high light stress. Although this includes strategies for adaptation, our explanation of the specific assembly steps withing PSII damage, the role of additional auxiliary proteins in photosystem remodeling or quality control, and degradation mechanisms remain largely unknown. Other future studies are necessary to address these missing elements that are involved in the responses of the plants, under high light and low light stresses. Medium level light, while less damaging than fluctuating high lighting levels will still have dramatic effects on many aspects of plant growth and development. State transitions and cyclic electron transport are examples of the mechanisms that plants use to help reduce damage from these changes in light. Over the past decade, research on how plants perceive and respond to changes in light has expanded considerably. Yet our knowledge is based mainly on controlled laboratory systems, meaning we lack a detailed understanding of how plants respond to the fluctuating solar resources they experience in real life. The sources of uncertainty include how plants perceive changes in light intensity, the cellular signaling pathways that mediate communication between the nucleus and organelles (including signals that alter photosynthetic systems to dissipate/rebalance excess excitation energy), as well as plastid-to-nucleus retrograde stress responses. More exploration is required to uncover all of the regulatory processes by which plants can cope with changes in light availability.

A better understanding of how plants handle light stress may give insight into ways we could us genetic manipulation to improve photosynthesis. This enables faster assembly or reassembly of photosynthetic machinery in response to changes in light intensity which, as the study shows, can be further facilitated by genetic modifications. Such an evolution enables better light energy utilization, reductions in damage to the photosystems, and higher performance of photosynthesis. Plants that are engineered to grow in controlled environments, such as in plant factories, could benefit from reduced acclimation requirements, thus allowing the plants to maintain photosynthetic efficiency at optimum levels without the need for frequent adjustments.
